# Sleeping Rooms

**Published:** 1872-12

**Authors:** 


### SLEEPING ROOMS

Are occupied a full third of a man's entire existence, and upon his breathing a pure or impure air for so large a portion of his life, the question of health and disease largely depends. The healthiest chamber is that, the windows of which face the south, so as to have the pure and warming and drying sunlight, for the greater part of the twenty-four hours.

Plastered walls are far the best, especially if the sides and ceiling are well whitewashed once a year. About May is the best for this, as the warm weather promotes the exhalation of odors which the lime has a tendency to absorb or neutralize.

This whitewash should not be tinted with any color. It has been noticed that in two adjoining rooms, where a number of girls were at work from month to month, those in the apartment tinted with yellow ochre complained of pain in the eyes and forehead, were often dull, dispirited, and melancholy, and were frequently unable to work.

In the other room, which was simply plastered, the workers seemed to be always cheerful and healthy. The ochre was removed, a pure whitewash was substituted, with a prompt return to cheerfulness and health.

If walls are painted, even of a white color, the particles of lead will in time become dry, and float off, and be breathed into the lungs.

All glazed wall-paper contains a large amount of lead or arsenic, sometimes both, and constantly throws off poisonous particles. Even if the paper is unglazed and contains no arsenic, it often happens that new paper is pasted on old, making it certain that obscure disease will result from the decay of the inner layer, which gives out emanations, poisonous enough in some cases to cause typhoid fever in a few nights. As a means of health, the breathing of a pure air during sleep is scarcely second to any other consideration, essential alike to old and young, to the feeble and the strong.

